# Gullivers Reise in die Psychose – geleitet von Jonathan Swifts eigenen Erfahrungen

**DOI:** 10.1007/s40211-025-00554-2

**Published:** 2025-10-23

**Authors:** Hans Förstl

**Affiliations:** Institut für Geschichte und Ethik der Medizin, Technische Universität München, Ismaningerstr. 22, 81675 München, Deutschland

**Keywords:** Calentures, Katatonie, Lilliput-Halluzination, Maritime Poriomanie, Skorbut, Thiamin-Mangel, Beriberi, Calentures, Catatonia, Lilliput-hallucination, Poriomania (maritime), Scorbutic Nostalgia

## Abstract

**Hintergrund:**

Jonathan Swift (1667–1745) besass ein anhaltendes Interesse an psychischen Störungen, die er häufig für satirische Zwecke einsetzte. Er entwarf 1704 eine Typologie verschiedener Geisteskrankheiten, wurde 1714 zum Vorsteher von Bedlam gewählt und verfügte bereits 1731, sein Erbe solle der Errichtung eines ersten Nervenkrankenhauses in Irland gewidmet werden.

**Fragestellung:**

Identifikation neuropsychiatrisch relevanter Inhalte von *Gullivers Reisen*.

**Material und Methode:**

Die literarische Quelle wurde systematisch und anhand der einschlägigen wissenschaftlichen Literatur aus Medizin und Geisteswissenschaften evaluiert.

**Ergebnisse:**

*Gullivers Reisen* – in erster Linie eine zeitkritische, politische Satire – wurde vor 300 Jahren veröffentlicht. Die Abenteuer des rastlosen Schiffsarztes Lemuel Gulliver auf verschiedenen entlegenen Inseln können als Reise in eine Psychose mit charakteristischen motorischen, sensiblen und kognitiven Störungen gelesen werden. Bei seiner Rückkehr nach England lehnt ein autistischer Gulliver den Kontakt mit Menschen einschliesslich seiner Familie ab und zieht die Gesellschaft von Pferden vor. Seeleute und Anstaltsinsassen waren zu Gullivers Zeiten ähnlichen somatischen Risikofaktoren ausgesetzt, die eine organische Mitursache damals beobachteter psychischer Störungen nahelegen (*scorbutic nostalgia*).

**Schlussfolgerungen:**

Bei retrospektiven Diagnosen ist grundsätzlich Vorsicht angebracht und dies gilt auch für fiktionale Figuren wie Lemuel Gulliver. Gäbe es keine ernsthaften Hinweise auf die Bedeutung besonderer somatischer Risiken auf den Schiffen und in den Anstalten jener Zeit – v. a. Hypovitaminosen –, läge der Eindruck nahe, Swifts Gulliver habe schon lange vor der medizinischen Wissenschaft eine Reihe von akzessorischen und Grundsymptomen der Schizophrenie beschrieben.

## Abstract (en)

**Background:**

Jonathan Swift (1667–1745) had long been interested in mental disease, which he also (ab)used for satirical purposes. He characterized various forms of madness in 1704, was elected governor of Bedlam in 1714, and in 1731 decided to dedicate his fortune to the founding of a first lunatic asylum in Ireland.

**Objective:**

To identify and consider the nature of neuropsychiatric features described in *Gulliver’s Travels*.

**Material & methods:**

Literary works by and biographies of Jonathan Swift were examined together with the pertinent medical and historical specialist literature.

**Results:**

*Gulliver’s Travels*, primarily a political satire, was published 300 years ago. The adventures of the restless naval surgeon Lemuel Gulliver on various remote islands can be understood as a journey into psychosis with characteristic motor, sensory and cognitive features. After his return home an autistic Gulliver refuses contact with his own species, the *Yahoo*-like humans, including his family and prefers the company of horses. Seamen and *lunatic* inpatients apparently shared a number of environmental risk factors and features of *scorbutic nostalgia*.

**Conclusion:**

Caution is advised when making a retrospective diagnosis, even with a fictional character like Lemuel Gulliver. If there were no hints at relevant organic causes—primarily hypovitaminoses—, one might suggest that Swift/Gulliver reported a number of characteristic symptoms of schizophrenia long before they were described in the medical literature.

## Hintergrund

Jonathan Swift sammelte im Laufe seines Lebens einige Erfahrungen mit neuropsychiatrisch bedingten Störungen. Swifts Vater Jonathan sen. (1640–1667) verstarb etwa zwei Monate nach der Zeugung von Jonathan jun. (1667–1745) und zwar vermutlich an einer Syphilis [82]. Onkel Godwin Swift (1628–1695), bei dem Jonathan in Dublin aufgewachsen war, entwickelte im Alter schwere Gedächtnisstörungen [57, 78]. Jonathan Swift selbst litt seit 1690 und vermehrt nach 1710 unter rezidivierendem, anfallsartigem Schwindel und Tinnitus, aller Wahrscheinlichkeit nach aufgrund eines Morbus Meniere [31, 57]. Diese Erfahrungen können zum nachhaltigen Interesse des Satirikers und anglikanischen Geistlichen an Nervenkrankheiten beigetragen haben. Im *Märchen von einer Tonne* (*Tale of a tub*) entwarf Swift 1704 eine knappe Skizze von Geistesstörungen, die man in Bedlam besichtigen konnte und die unter anderem Kant bei seinem *Versuch über die Krankheiten des Kopfes* inspirierte [31]. Um psychisch Kranken zu begegnen, musste man sie jedoch nicht in *Bedlam* besuchen, wo damals nur die voraussichtlich Heilbaren zeitweise Aufnahme fanden; elende *Lunatics* irrten durch die Straßen des ganzen Königreiches, in London wie in Dublin [1, 75]. 1714 wurde Swift zu einem der Vorsteher (*governors*) von Londons *Bedlam* gewählt. 1721 wurde Swift als Treuhänder von Dr. Richard Steevens Vermögen zur Errichtung eines Krankenhauses bestellt; 1725 als Vorstand von Dublins Findlingshospital sowie des Armen- und Arbeitshauses; 1735 als Sachwalter um mit dem Vermögen von Mrs. Mary Mercer ein Hospital für chronische Erkrankungen zu bauen [27, 82]. Sein gesamtes eigenes Vermögen (bei seinem Tod 12.000 £) versprach er bereits 1731 der Errichtung einer ersten psychiatrischen Klinik in Irland zu widmen und zwar mit der Begründung, kein anderes Land benötige eine derartige Institution so sehr [27, 31]. Dublins *St. Patrick’s Hospital for the Imbeciles* wurde 1757 zunächst für unheilbar psychisch Kranke eröffnet und existiert bis heute als psychiatrische Klinik [75].

Swift wählte für *Lemuel Gulliver* einen sprechenden und dabei vieldeutigen Namen (hebr. *Lemu’el* = gottergeben; engl. *to gull*/*the gull *Angeber und Betrüger, aber auch: dummes Tier) [42, 46] sowie die Perspektive eines Ich-Erzählers, der selbst berichtet wie er die Welt wahrnimmt und erinnert. Es ist anzunehmen, dass Swift den Schiffsarzt Gulliver nicht ohne Absicht als Protagonisten der umfangreichen Reiseberichte aus fernen Ländern wählte, sondern damit bewusst einen medizinischen Bezug herstellte. Jonathan Swift wandte einige Raffinesse auf, um den fiktiven Gulliver als vermeintlichen Autor seiner politischen Satire erscheinen zu lassen [10, 25, 53].

Dieser Beitrag beschäftigt sich mit der Frage, wie viel neurologisch-psychiatrische Erfahrung in die Erzählungen von Swift/Gulliver eingeflossen sein kann und welche Ursachen für derartige Störungen unter den damaligen Bedingungen möglicherweise eine besondere Rolle spielten. Die folgenden Punkte werden angesprochen:

- Welche neuropsychiatrischen Symptome werden in Gullivers Reisen dargestellt?
- Welchen Risiken waren Seeleute auf langen Reisen oder auch Insassen von Asylen und damit der fiktive Gulliver ausgesetzt?
- Wie verlief die Karriere des Protagonisten selbst? Kann es sich dabei um eine in Literatur verwandelte Krankengeschichte handeln?

## Methode

Der Reisebericht von Lemuel Gulliver wurde nach historisch-kritischen Ausgaben [85] hinsichtlich potentiell neuropsychiatrischer Auffälligkeiten untersucht. Zusätzlich wurden mehrere Biographien Jonathan Swifts (z. B. [26, 82]), Kommentare zu Swifts Werken [19, 25, 27, 33, 36, 53, 66, 67, 72, 74, 78, 85, 90] sowie Sekundärliteratur aus Anglistik und Medizin einschliesslich Medizingeschichte durchsucht [Stichwortsuche nach „Jonathan Swift“, „Gulliver“, „Lilliput“ etc. in PubMed, ISTOR, Web of Science, Google Scholar] und als Quellen herangezogen. Historisch-politische Bezüge werden weitgehend ausgespart, ebenso wurde aus Gründen des Umfangs auf die Beschreibung und Beurteilung einiger Abstecher auf noch weiter entlegene Inseln verzichtet.

## 1. Eigenanamnese (stark gekürzt nach den Berichten Gullivers)

### Vorgeschichte

Lemuel Gulliver wurde als dritter von fünf Söhnen in einfachen Verhältnissen unweit von Nottingham geboren, besuchte ab 1675 das Emmanuel College in Cambridge und arbeitete ab 1678 vier Jahre lang als Gehilfe des Wundarztes *Master Bates* in London [85, I.1]. Daneben habe er sich die Grundlagen der Mathematik und der Navigation angeeignet. Danach sei er zum Studium der Medizin zwei Jahre und sieben Monate nach Leyden gegangen. *Master Bates* habe ihn dann als Schiffschirurgen empfohlen und so sei er während der nächsten drei Jahre auf der *Swallow *ein oder zweimal in die Levante und in einige andere Gegenden gefahren. Dann habe er beschlossen sich in London in der *Old Jewry* als Arzt niederzulassen. Ihm sei geraten worden zu heiraten und Mrs. Mary Burton habe 400 £ mit in die Ehe gebracht. Auch aufgrund mangelnder Beziehungen habe seine Praxis zu wenig eingebracht und er habe beschlossen, wieder zur See zu fahren. So habe er in den nächsten sechs Jahren auf zwei Schiffen Reisen in die Karibik und nach Fernost unternommen. Wieder zurückgekehrt, sei er in die *Fetter Lane* umgezogen und von da nach *Wapping* in die Nähe der Werften, aber innerhalb der nächsten drei Jahre hätte seine Arbeit nicht genug eingebracht, also habe er wieder als Schiffsarzt angeheuert.

Nebenbei betont Gulliver auch sein exzellentes Gedächtnis, seine Sprachbegabung und die Verwandtschaft zu seinem Cousin *William Dampier* (1651–1715), dem berühmten Forschungsreisenden, mehrfachen Weltumsegler und Freibeuter [20].

### Lilliput und die Lilliputaner

**Abb. 1:**
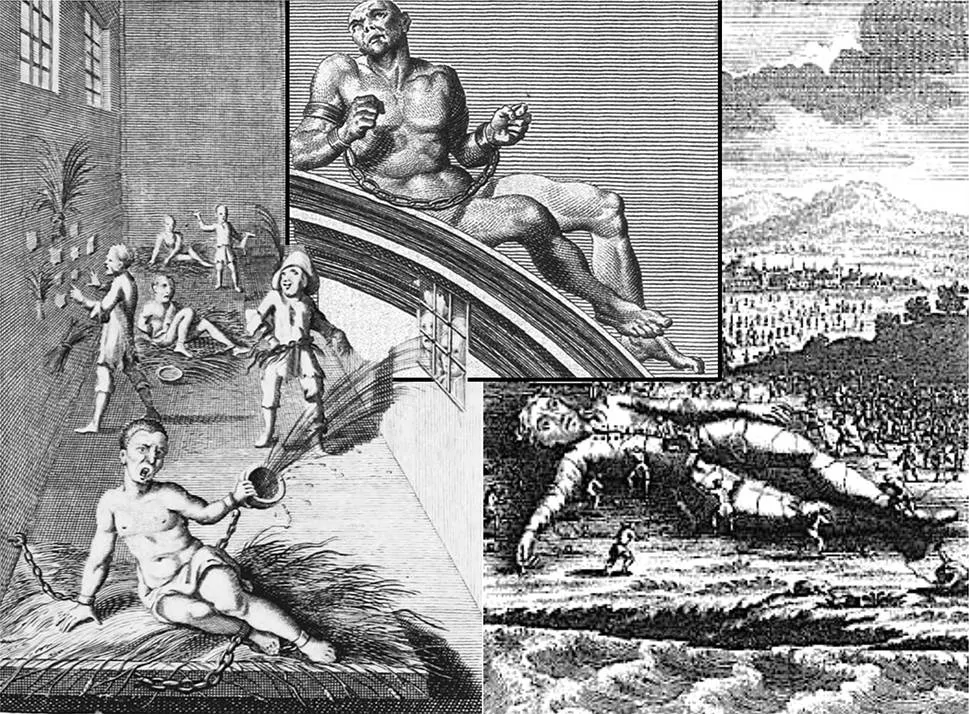
Oben *Der Rasende *(*raving madness*), Stich eines unbekannten Künstlers nach Caius Cornelius Cibbers (1630–1700) Steinfigur über dem Eingang zu Bedlam. *Links* *unten* Illustrationen aus Jonathan Swifts Schilderung der Zustände in Bedlam aus dem *Märchen von einer Tonne* (*Tale of a* *Tub*; 5. Aufl., 1710; Bernard Lens III., 1682–1740). *Rechts* *unten* eine erste Darstellung des gefesselten Helden, die sich erstmals in der zweiten französischen Ausgabe von Gullivers Reisen findet (*Les voyages* de *Gulliver*; 2. Aufl., Paris, 1727; unbekannter Künstler). Ähnlichkeiten in der Bildgestaltung werden in der Folge beibehalten und dem Zeitgeschmack angepasst. Gullivers Fixierung bis zur Bewegungsunfähigkeit ist das am weitesten bekannte und am besten erinnerte Bildmotiv aus allen seinen Abenteuern [66, 85]

Auf dem Weg in die Südsee gerät die *Antelope* am 5. November 1799 vor Australien in Seenot (Tab. 1). Gulliver erwähnt den schlechten Zustand der Mannschaft; die Männer seien durch Strapazen und schlechte Ernährung stark geschwächt gewesen und zwölf Seeleute waren bereits verstorben. Er habe an Bord noch ein halbes Pint (ca 300 ml) Brandy getrunken (ca. 50 %), bevor er mit anderen verzweifelt versuchte das Schiff rudernd freizuschleppen. Der Versuch misslang, das Ruderboot kenterte und er schwamm lange, ehe er gegen Abend Boden unter den Füssen spürte. An Land schlief er entkräftet ein und als er am nächsten Morgen erwachte, fand er sich an den Boden gefesselt (Abb. 1), war umgeben von zahlreichen, etwa 15 cm grossen Wichten, die auf ihn einredeten, ihn bald mit hunderten von Pfeilen beschossen, die Blasen auf Gesicht und Hand verursachten usw. ehe man sich in der Folge zeitweise auf ein einigermassen gütliches Auskommen verständigte (aufgrund des erheblichen Alkoholgenusses am Vortag ist zu berechnen, dass er beim Erwachen noch Restalkohol im Blut hatte). Der Wein, den er dort später aus Fässern trank, sei besser gewesen als jeder Burgunder. Als er eine Feuersbrunst mit seinem Urinstrahl löschte, hatte er davor besonders viel konsumiert. Der Rest dieser Geschichte darf als bekannt vorausgesetzt werden.

**Tab. 1:** Gullivers Reisedaten (Abfahrt/Ankunft/Rückkehr), Hinweise auf Symptome mit potentiell neuropsychiatrischer Relevanz und auf zugrundeliegende oder begleitende somatische Faktoren von Gulliver und Mitreisenden (zu *ague* und *calentures* siehe Text; EPMS = extrapyramidalmotorische Störungen) [85].

*Reisedaten*	*Somatische Faktoren* *und Symptome*	*Neuropsychiatrie,* *Verdacht auf*
*Vorgeschichte*		Grössenideen; Abstammungswahn
*04.05.1699 Bristol* *06.11.1699 ***Lilliput** *13.04.1703 Downs*	Mangelernährung; Überanstrengung; Erschöpfung; hoher Krankenstand; Alkohol; juckende Efflorenzenzen	Akinetisch-rigid oder katatone Symptome (DD: pathologisches Erwachen); Lilliput-Halluzinationen; Körper- und Geschmackshalluzinationen; Neologismen, verschrobene Sprache; Konfabulationen
*20.06.1703 Downs* *17.06.1703 ***Brobdingnag** *03.06.1706 Downs*	*Ague*; Gewichtsverlust	Angst, Verletzlichkeit, Ekel; Gulliver-Halluzination; motorische Manierismen (oder EPMS)
*05.08.1706 Downs* *xx.yy.1707 ***Laputa** *10.04.1710 Downs*	Hoher Krankenstand; Erschöpfung	Transitivismus; Akathisie, okulogyre Krise, Dyskinesie; reduplikative Paramnesie
*07.11.1710 Portsmouth* *09.05.1711 ***Yahoos et al.** *05.12.1715 Downs*	Tropendelir (*Calentures*)	Motorische Manierismen (oder EPMS); Dolittle-Phänomen; Misanthropie, Ekel vor Menschen und sich selbst

Zurück in London hielt er es nur zwei Monate bei der Familie aus, ehe ihn erneut die unstillbare Unruhe überkam. Von einem Sohn Jonny war kurz zu erfahren, dass er in die Grundschule geht. Tochter Betty sei verheiratet und habe jetzt selbst Kinder.

### Brobdingnag und die Riesen

Die Reise war wegen einer *Ague* (siehe unten) des Kapitäns unterbrochen worden, aber im Juni 1703 landet Gulliver an einer nordwestlichen Landzunge Amerikas. Seine Kameraden sichten die riesigen Einwohner vor ihm und lassen ihn allein zurück. Alles ist monströs und überaus anstrengend. Für Geld wird er zur Schau gestellt. Der Angriffe von Ratten, und auch von Fliegen kann er sich kaum erwehren. Gefahren lauern überall. Die Stimmen, die grobporige Haut der Riesen, ihre Gerüche, Körperfunktionen und Krankheiten erschrecken und ekeln ihn an [87]. Er magert zum Skelett ab. Die riesige Königin meint es eigentlich gut mit ihm und rät zu mehr Bewegung, er aber fühlt sich hilflos und wird melancholisch. Die Sprache der Riesen erlernt er schnell, aber ihren Respekt erwirbt er nicht, sondern wird nachts wie ein Spielzeug in eine Schachtel gesperrt. Ortsansässige Experten halten ihn für ein mickriges Insekt, ein blosses Experiment der Natur.

Gerettet und daheim in London fürchtet er auf die winzigen Menschen zu treten, beugt sich zu ihnen herab, wenn er mit ihnen spricht, bückt sich tief ehe er durch eine Tür geht und nimmt ansonsten seine steif aufgereckte Haltung ein, mit nach oben gerichtetem Blick. Andere meinen, er hätte seinen Verstand verloren. Nach zehn Tagen wird er zu einer neuen Ausfahrt eingeladen.

### Laputa und die Geistesmenschen

**Abb. 2:**
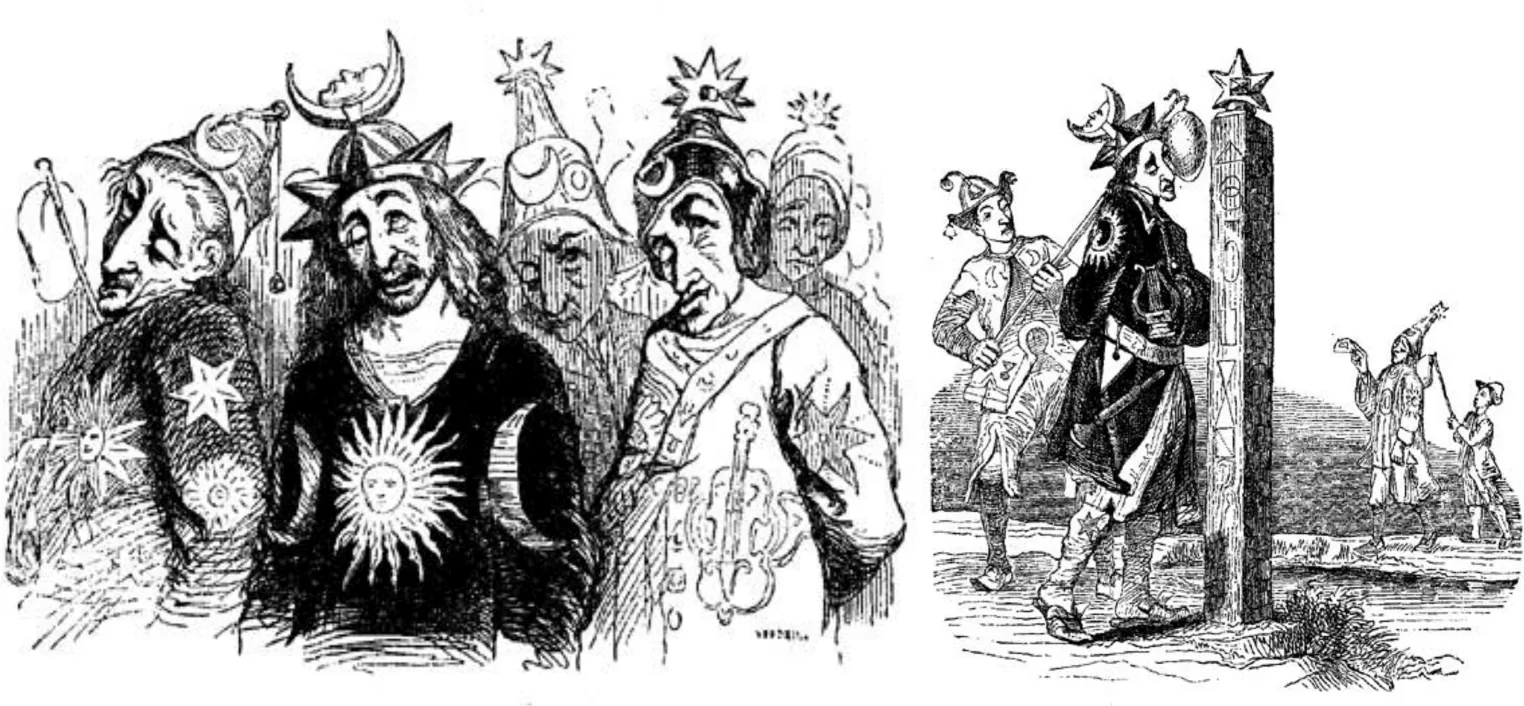
Die Bewohner Laputas mit Akathisie-, Blickkrampf- und Dyskinesie-ähnlichen Merkmalen und ihre Aufpasser (*Climenolen*), die durch ein Betupfen mit Ballons ein wenig Aufmerksamkeit herstellen um die Laputianer auf die richtige Bahn zu lenken. Die Darstellung Jean-Jaques Grandvilles (1803–1847) aus dem Jahr 1838 ist sehr nah an Swifts Text und an einer traditionellen Ikonographie von Geisteskrankheiten mit mimischen und anderen psychomotorischen Symptomen [85]

Auf dem Weg nach Fernost muss sich die Mannschaft bereits auf einer Zwischenstation in Madras drei Wochen lang erholen; viele sind krank. Danach verschlägt es Gulliver von Tonkin aus durch Sturm, Piratenüberfall und nach langem Rudern auf eine Insel über der eine andere, kleinere magnetisch schwebt. Zu diesem *Laputa* wird er hochgezogen und befindet sich inmitten eines ebenso chronisch vergeistigten wie zerstreuten, ganz der Mathematik, Astronomie und Musik hingegebenen Hofstaates. Die Männer sind ungeschickt, verlangsamt und jederzeit perplex, wenn es um die Dinge des Alltags geht, dabei ständig angespannt und ängstlich unterwegs. Den Kopf nach hinten oder zur Seite gelegt, ein Auge auf den Zenith, das andere nach innen schielend, müssen sie ständig von Wärtern (*Climenolen*; z. B: lat. *clinamen*, Ablenkung bei gerader, autistischer Bewegung durch den Raum) [31] begleitet werden, die durch sanftes Beklatschen mit einem Ballon von Ohren, Mund oder Augen ein Minimum an Kommunikation und Aufmerksamkeit herstellen (Abb. 2). Diese Männer sind nicht kontaktfähig und nehmen sowohl Gulliver, als auch ihre eigenen Frauen kaum wahr. Er fühlt sich geringgeschätzt und vernachlässigt.

Die Rückfahrt gelingt nach einigen Abstechern (die hier ausgespart werden). Kapitän Mendez (engl. *to mend*, helfen etc) [42]. nimmt Gulliver in Japan an Bord und bringt ihn nach Amsterdam. Unterwegs versterben nur drei Seeleute an Krankheiten, ein vierter stürzt vom Fockmast zu Tode.

### Houynhnhmns und Yahoos

Diesmal hält es Gulliver ganze fünf Monate zuhause aus, ehe er – nun als Kapitän – wieder in See sticht. Da auf dem Weg in die Karibik mehrere Seeleute Opfer eines Tropendelirs (*Calentures*) [42, 59] werden, nimmt er dort neue Männer an Bord, bei denen es sich allerdings um Piraten handelt. Sie setzen ihn zunächst in seiner Kabine fest, während auf der Fahrt nach Madagaskar weitere Seemänner ihren Krankheiten erliegen. Gulliver wird auf einem Eiland ausgesetzt und begegnet dort behaarten, groben, aber äusserlich menschenähnlichen, grimassierenden, aggressiven, wilden und damit in ihrem Gebaren fremdartigen Wesen, den *Yahoos* (engl. *you; who* = sinngemäss z. B. wer bist Du) [42]? , die ihn auf übelste Weise beschmutzen (Diese *Yahoos* stimmen in ihren anatomischen Merkmalen weitgehend mit Dampiers Schilderung australischer Ureinwohner überein) [20]. Beherrscht werden sie und das Land von edlen Pferden, den *Houynhnhmns* (lat. *homo sapiens*; und onomatopoetisch für Pferdeschnauben), die keine Krankheit kennen. Gulliver erstattet den bewunderten Pferden einen recht kritischen Bericht über die Gebräuche und Gebrechen Europas, wo es sogar einen Berufsstand gebe, der sein Geld mit den Krankheiten anderer Leute verdiene. Er fühlt sich wohl im Kreise der gebildeten *Houynhnhmns*, sogar deren Gang und Gesten werden ihm zur Gewohnheit.

Am Ende wird er jedoch als primitiver *Yahoo* des Landes verwiesen und gelangt über Australien auf einem portugiesischen Schiff nach Lissabon und schliesslich nach England.

### Heimkehr

Am 5. Dezember 1715 landet Gulliver um neun Uhr morgens an der Spitze Südostenglands und gelangt um drei Uhr am Nachmittag desselben Tages zuhause in Rotherhithe an. Sein Ekel vor den *Yahoos* als abscheulichen Varianten des Menschlichen war in keiner Weise von einer gegenseitigen Sympathie mit den distanzierten *Houyhnhnms* kompensiert worden. Dennoch ist Gulliver als autistischer Misanthrop zurückgekehrt. Selbst seine eigene Familie ist ihm widerlich geworden. Er erwirbt als erstes zwei Hengste, mit denen er sich täglich mindestens vier Stunden unterhält.

Seinen Reisebericht verfasst Gulliver laut Swift ab 1720. Am 28. Oktober 1726 wird die erste Auflage von *Gullivers Reisen* veröffentlicht und am 2. April 1727 schreibt Gulliver angeblich noch einen Brief an den Herausgeber namens Sympson. Danach verlieren sich seine Spuren.

## 2. Symptome und Diagnosen

Geschildert werden auf Gullivers Reisen einige medizinische Probleme, die erklärungsbedürftig sind, und Symptome, die mit einiger Vorsicht diagnostisch zugeordnet werden können (Tab. 1).

### Historische Erkrankungen

Zu den erklärungsbedürftigen somatischen Erkrankungen aus Gullivers Tagen gehört die *Ague*, Oberbegriff für ein rezidivierendes Fieber, bei dem es sich häufig um eine mildere Verlaufsform der Malaria, hervorgerufen durch *Plasmodium vivax*, handelte, die damals in den englischen Marschen endemisch und damit auch bei englischen Seeleuten häufig war [24, 41, 42].

Die *Calentures* (span. *Calentura*, Fieber) waren ein seltsames, aber keineswegs seltenes, sondern angeblich weit verbreitetes, halluzinatorisches Fieberdelir, das nur in tropischen Breiten auftrat, bei dem Seeleute glaubten Land vor sich zu sehen und über Bord sprangen [42, 59]. Noch Bernhard Nocht [64] berichtete von der besonders hohen Suizidrate der Heizer (*Feuerleute*) auf Schiffen am Ende des 19. Jahrhunderts. Eine Untersuchung der *UK National Union of Seamen* über Todesfälle in der britischen Handelsmarine zwischen 1964 und 1978 führte immer noch 577 von insgesamt 3778 Todesfällen auf Halluzinationen zurück, bei denen die Seeleute vorher auffallend lange auf den Horizont starrten [51].

### Psychopathologie

Der rastlose Gulliver zeigt durchgehend Züge einer maritimen Poriomanie, einer Wandersucht zu Wasser. Das weite Konzept der Poriomanie umfasst psychogene, Substanz-induzierte, organische und katatone, aber auch Persönlichkeits- und Verhaltensstörungen ([3]; zur terrestrischen Poriomanie siehe [35]). Daneben liefert Gulliver durch den Verweis auf Vetter Dampier Anhaltspunkte für einen möglichen Abstammungswahn [6]. Auch die mikroptischen *Lilliput-Halluzinationen* [17, 55] und die makroptischen *Gulliver-Halluzinationen* [28] sind ätiologisch vieldeutig und können auftreten im Zusammenhang mit Angst und dissoziativen Störungen [47, 79], Intoxikationen und Delir [7, 89], Migräne, Narkolepsie oder zerebralen Anfällen [7, 63], strukturellen Erkrankungen von Augen und Gehirn [7, 16]. Bei seiner letzten Reise und der Heimkehr beschreibt Gulliver ein Dolittle-Phänomen mit scheinbar verständlichen, halluzinierten Tierstimmen [22]. Die Schilderung der notorisch geistesabwesenden Laputianer und ihrer Aufpasser legt den Verdacht nahe, Gulliver berichte nicht seine Erlebnisse aus der Südsee, sondern aus einem Londoner Tollhaus (Tab. 1, Abb. 1, Abb. 2). Dabei könnte es sich um eine wahnhafte Verwechslung des Ortes handeln, eine reduplikative Paramnesie [32, 70], gepaart mit einer möglichen Verkennung der Rollen: nicht Gulliver ist der Arzt, sondern ein Kranker, der alle anderen für geistesgestört hält [81]. Dies alles wird mit grosser Intensität zusammenfabuliert [48, 49, 77]. Insgesamt lässt sich keine einzelne spezifische Erkrankung auf Anhieb dingfest machen, aber eine rätselhafte Wahnstimmung scheint Gullivers Reisen von Anbeginn zu durchziehen.

### Bewegungsstörungen

Ob es sich bei Gullivers initialer Immobilität in Lilliput um einen katatonen Zustand, ein abklingendes Delir oder einen hochgradigen Erschöpfungszustand mit Restalkohol im Blut handelte, oder um alles zusammen, muss letztlich offen bleiben [38, 45, 61] (Abb. 1). Die Symptomschilderung der Bewohner Laputas erinnert an die klinischen Bilder von Akathisie [37], Dyskinesien und okulogyren Krisen [4, 35, 60] (Abb. 2). Vor dem Zeitalter von Enzephalitis lethargica und von Neuroleptika muss bevorzugt an andere Formen und Ursachen der Dyskinesien gedacht werden wie psychogene Störungen, anhaltende Tics oder Strabismus [60, 69]. Gullivers eigener Gebrauch unbekannter Sprachen (Neologismen) und seine motorischen Manierismen können weniger als genuin extrapyramidalmotorische Störungen [12, 71], denn als dysfunktionale Adaptionsversuche an eine fremde Umgebung oder als Hospitalisierungsfolgen aufgefasst werden.

### Mortalität auf See

Die Wahrnehmungen und Berichte der Reisenden waren aller Wahrscheinlichkeit nach mitbeeinflusst von einigen Umständen, die heute teilweise überwunden und vergessen sind. Die Verlustraten an bei der Ausfahrt auf engstem Raum zusammengepferchter Seeleuten waren hoch: William Dampier brachte 1711 nur 18 von 183 und George Anson 1744 nur 145 von 1955 Männern von ihren Weltumsegelungen zurück [20, 31, 59, 85]. Schiffe sanken, Kranke starben oder wurden zurückgelassen, andere flohen oder verloren ihr Leben bei Unfällen. Zur Vulnerabilität trugen die mangelhafte Hygiene bei, Infektionen, stark gepökelte oder verfaulte Lebensmittel voller Maden, Rüsselkäfern und Rattenharn, schmutziges Trinkwasser und verdorbenes Leichtbier. Hochprozentiger Alkohol war bei längeren Ausfahrten das einzig hygienisch unbedenkliche – und dabei neurotoxische – Getränk an Bord.

### Speisepläne an Bord

Die Seeleute hatten seit Mitte des 17. Jahrhunderts ein Anrecht auf eine Gallone Dünnbier (4,5 l) und ein Pound Kekse pro Tag (450 g); vier Pounds gepökeltes Rindfleisch, zwei Pounds Schweinefleisch, drei Achtel Fisch, ein Quart Erbsen (1,15 l), sechs Ounces Butter und zwölf Ounces Käse pro Woche (1 Ounce = ca. 28 g). Im Mittelmeer und in Übersee ersetzten Wein das Bier, Rosinen das Rindfleisch, Reis oder Stockfisch den Kabeljau und Olivenöl Butter oder Käse [21]. Der Wochenplan (*regulations and instructions*) der britischen Admiralität stammt aus dem Jahr 1733; zusätzlich bekam jeder Mann noch maximal ein halbes Pint Essig pro Woche. Die tägliche Kalorienmenge erreichte 5000 kcal ([58]; Tab. 2). Es gab aber auch Strecken auf denen die Matrosen über lange Zeit kaum zu essen bekamen und sich über viele Tage weigerten, das Brackwasser zu trinken [20].

**Tab. 2:** Speisepläne von Seeleuten der britischen Marine und Patienten in Bedlam: Dünnbier, Getreide- und Milchprodukte sowie Fleisch standen auf See und in der Anstalt im Vordergrund; frisches Gemüse und Obst fehlten [1, 21, 58].

	Marine	Bedlam
*Montag*	Dünnbier; Kekse, Haferbrei, Käse, Butter	Dünnbier; Brot, Erbseneintopf oder Brei, Käse oder Butter
*Dienstag*	Dünnbier; Kekse, Rind	Dünnbier; Brot, Hammel oder Kalb, Brühe
*Mittwoch*	Dünnbier; Kekse, Erbsen, oder Haferbrei, Käse, Butter	Dünnbier; Brot, Milcheintopf oder Brei, Käse, Butter
*Donnerstag*	Dünnbier; Kekse, Schwein, Erbsen	Dünnbier; Brot, Rind, Brühe
*Freitag*	Dünnbier; Kekse, Erbsen, Haferbrei, Käse, Butter	Dünnbier; Brot, Milcheintopf oder Brei, Käse, Butter
*Samstag*	Dünnbier; Kekse, Rind	Dünnbier; Brot, Erbsenbrei, Milchreis, Käse oder Butter
*Sonntag*	Dünnbier; Kekse, Schwein, Erbsen	Dünnbier; Brot, Rind, Brühe

### Speisepläne im *Tollhaus*

Wie bei der Marine, wechselten auch in Bedlams Wochenspeiseplan aus dem Jahr 1677 Fleischtage mit fleischfreien Tagen. Auch hier war die Diät von Getreide- und Milchprodukten bestimmt und es gab Fleisch. Die Mengen waren nicht so genau festgelegt und es ist davon auszugehen, dass die Rationen weit kleiner bemessen waren, zumal führende Mediziner (z. B. Robert Burton, Thomas Willis, Richard Mead, William Battie u. a.) über lange Zeit die Position vertraten, eine schlanke Diät sei geeignet wieder Vernunft einkehren zu lassen. Früchte der Saison waren gelegentlich erlaubt, würden aber wegen der Fäulnisgefahr äusserst zurückhaltend eingesetzt [1]. Damit war der Mangel an Vitamin C und anderen Vitaminen der wesentliche gemeinsame Nenner dieser Ernährung zur See und im Asyl [30, 31].

### Mortalität an Land

**Abb. 3:**
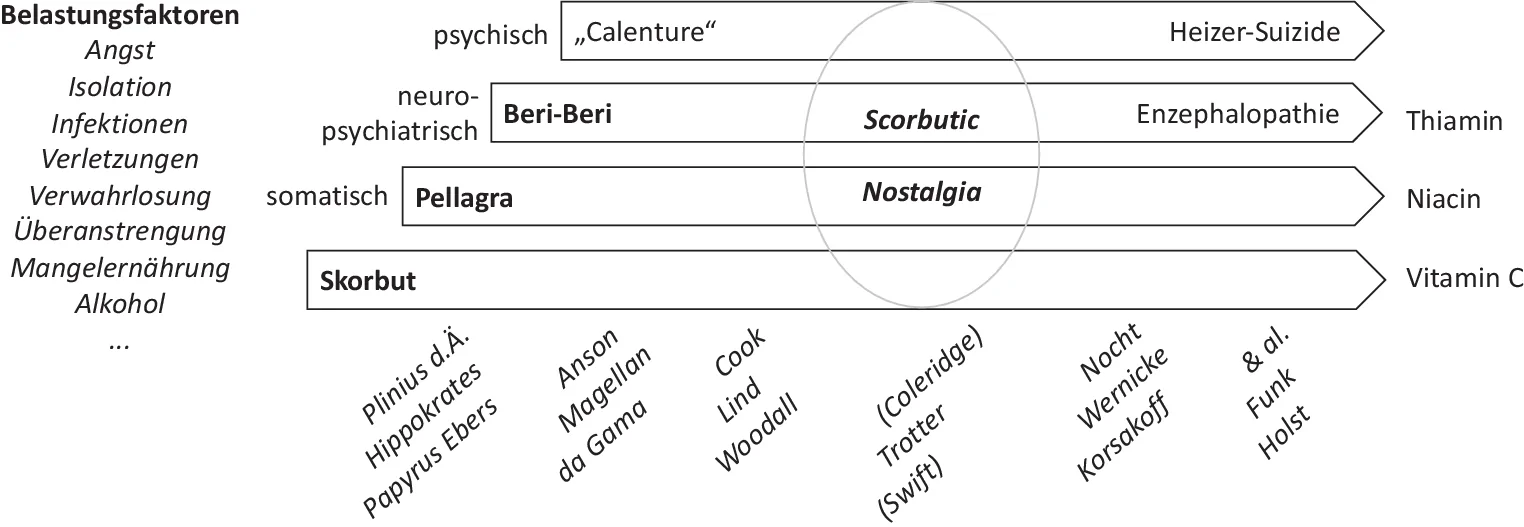
Symptome des Skorbut (Schaarbock, engl. *scurvy*) wurden vermehrt zu Zeiten grosser Feldzüge beschrieben und vor allem im Zeitalter weiter Seefahrten, die länger als siebzig Tage dauerten. Das Befundmuster wurde von ebenfalls lange bekannten Krankheiten mit teilweise gemeinsamen Risikofaktoren überlagert: Pellagra, Beri-Beri und Halluzinosen, die vor allem in heissen Klimaten beobachtet wurden (*Calentures*). Die *Scorbutic Nostalgia* mit einer Kombination somatischer und neuropsychiatrischer Symptome beschrieb Thomas Trotter erst 1792 [86]. Weder lange bekannte Erfahrungen aus der Volksmedizin, noch erste wissenschaftliche Erkenntnisse, wie z. B. von James Lind, fanden raschen Eingang in die Vorbeugung und Behandlung. Bernhard Nocht bekannte noch 1906 und damit wenige Jahre vor der Definition der Vitamine durch Funk, Holst und andere, dass die eigentlichen Ursachen von Beri-Beri und Skorbut nicht bekannt seien [13, 39, 50, 52, 64]

Metzger [62] schrieb 1784 über seine Inspektion des Königsberger *Irrhaus*es: *Die Speisen welche den Elenden gereicht werden, entsprechen dem kläglichen Aufenthalt. … Hieraus erklärt es sich, warum alle unsere Wahnsinnigen einen Ansatz zum wahren Skorbut haben, auch mehrenteils an dieser Krankheit sterben. Die Luft in den Irrstuben ist so pestilenzialisch, dass nur die Blödsinnigen, welche man noch ein- und ausgehen lässet, einige Jahre im Irrenhaus aushalten. Die Eingesperrten sterben in Kurzem, bisweilen ein paar Tage nach ihrem Eintritt*. Die alarmierenden Sterberaten in den Einrichtungen für psychisch Kranke wurden in der ersten Hälfte des 19. Jahrhunderts Gegenstand regelmässiger statistischer Untersuchungen und damit Massstab der Versorgungsqualität [2, 29, 80]. Wie auf den Schiffen gewann auch in den Asylen die Versorgung mit Luft und Kalorien an Interesse, wobei man sich an den Empfehlungen Justus von Liebigs orientierte [76]. Erste wissenschaftliche Erkenntnisse zur Bedeutung der Vitamine folgten erst später (Abb. 3).

### Skorbut

Lange Zeit wurden am ehesten die schlechte Luft an Bord der Schiffe und die Ferne zum Festland (*earthsickness*) verantwortlich gemacht für Skorbut, hohen Krankenstand und hohe Sterblichkeit der Seeleute [15]. Weit mehr als 100 Jahre vor James Linds erster kontrollierter Studie zur Behandlung von Skorbut [30, 84, 88] waren sowohl in der Volksmedizin vieler Länder und in der Medizin die Bedeutung von frischem Gemüse und Früchten eigentlich gut bekannt [15, 30]. John Woodall beschrieb bereits 1617 die Erschöpfung (*lassitude*) als ein führendes Symptom des Skorbuts und empfahl dem Schiffsarzt ausdrücklich den Einsatz von Zitronensaft [93]. Samuel Johnsons Wörterbuch aus dem Jahr 1798 zitiert Swifts Freund Dr. John Arbuthnot bei der Charakterisierung des *Scurvy* als Erkrankung in kalten Ländern oder von Bewohnern des Marschlandes [42, 72]. Der Eintrag zu *Scurvy* wird im Wörterbuch alphabetisch gefolgt von *Scurvygrass* (*spoonwort*), also Löffelkraut, dessen Vitamin-C-Gehalt doppelt so hoch ist wie der von Citrusfrüchten. Erst James Cook nahm aber regelmässig frisches Gemüse und Früchte an Bord und gab seiner Mannschaft ausreichende Erholungszeiten [15, 30, 65] (Abb. 3).

***Scorbutic nostalgia*** wurde 1792 von Thomas Trotter beschrieben [51, 86]. Neben entzündeten Mündern, wunder Haut, erweiterten Pupillen, Doppelbildern, Tinnitus und überempfindlichen Nasen schilderte er die – bei grosser Erschöpfung – übersteigerte Wahrnehmung und Einbildungskraft mit extremer Schreckhaftigkeit. Das heftige Verlangen nach sauberer Nahrung, frischem Wasser, festem Boden unter den Füssen, Weite, Luft und der Heimat ging dabei über in Illusionen und Träume von grünen Wiesen und plätschernden Gewässern. Das Erwachen war schmerzhaft und der Enttäuschung folgten Stöhnen, Klagen und kindliches Weinen (Abb. 3) [59, 86].

Neuere Untersuchungen zeigen, dass Vitamin-C-Mangel heute neben Apathie, Angst, Depression, Reizbarkeit und kognitiven Defiziten auch zu extrapyramidalmotorischen Symptomen führen kann; aus Daten der letzten Jahrzehnte lasse sich aber kein eindeutiger Zusammenhang mit affektiven oder schizophreniformen Psychosen herleiten [12, 71]. Dies weicht von der historischen Quellenlage deutlich ab und das Bild der *Schaarbocks* war bis in 18. Jahrhundert deutlich psychopathologisch mitgeprägt [31, 88, 93].

### Stress und Komorbidität

James Cook erkannte nicht nur die Bedeutung frischer Nahrungsmittel zur Vorbeugung, sondern auch die zu hohe Arbeitsbelastung als Risikofaktor für die Entstehung des Skorbut [65]. Neben Folgen des Vitamin-C-Mangels wurden noch bis ins 20. Jahrhundert schwere B‑Hypovitaminosen auf Segelschiffen und Expeditionen beobachtet [34, 50]: Pellagra als Folge eines Niacin-Mangels und Schiffs-Beri-Beri durch Thiamin-Mangel, die von einer akuten Wernicke-Enzephalopathie – falls überlebt – in ein amnestisches Korsakoff-Syndrom übergehen konnten [39, 44, 48, 49, 52]; (Abb. 3).

Allgemeinen Erklärungsmodellen entsprechend werden Kompensations- und Anpassungsfähigkeit durch mehrere gleichzeitig einwirkende physische und psychische Belastungsfaktoren überstrapaziert [9]. Affektive und posttraumatische Belastungsstörungen werden nach Rettung aus akuter Seenot beobachtet [54], schwerwiegende kognitive Defizite durch Beri-Beri infolge entwickelten sich nach den grundsätzlichen Erkenntnissen über die Bedeutung der Vitamine [13, 39, 44] nur noch in länger anhaltenden und extremen Mangelsituationen wie Krieg und Gefangenenlager [8, 83, 91] (Abb. 3).

## Diskussion – Kontext und Karrieren.

### Historischer Kontext

Es fällt nicht leicht sich die Lebensbedingungen in Schiffsrümpfen und *Tollhäusern* zu vergegenwärtigen: Angst, Verzweiflung, Hilflosigkeit bei sehr schlechter Alimentierung und fehlender Hygiene – ganz zu schweigen von vorbestehenden Erkrankungen. Der Absturz in die Verwirrtheit und die Ausflucht in die Phantasie können nicht nur psychische Bewältigungsstrategien aufgefasst werden, denn der Weg dorthin war durch die organischen Veränderungen der Hirnfunktion gebahnt: zu wenig hochwertige Nahrungsmittel, kein sauberes Wasser, damit meist unzureichende Hydrierung und stattdessen auf See sporadisch Alkohol für die, welche ihn sich leisten konnten.

Wenige der fiktiven oder realen Überlebenden gelangten zu literarischem Ansehen von Homers Odysseus bis zu Coleridges dauerhaft delirantem *Ancient Mariner. *Selbst in Plinius naturkundlichen Schriften und die Berichte von Francis Drake, Walter Raleigh, William Dampier u. v. a. mischen sich Nachrichten über seltsamste Vorkommnisse und Lebewesen an den Rändern der bekannten Welt (z. B. [31, 90]). Daneben finden sich unterhaltsame und offensichtliche literarische Phantasieprodukte der Zeit wie Rabelais Riesen, Bergeracs Reise zum Mond und Voltaires *Micromegas* [23, 27]. Damit stehen Gullivers Berichte in einer langen Tradition von Abenteuern mit schillerndem Realitätsbezug, wobei jedoch seine Anmerkungen mit medizinischer Relevanz hervorstechen. Das Verhältnis von nah und fern, von gross und klein war spätestens mit den astronomischen Erkenntnissen von Kepler und Galilei sowie den mikroskopischen Entdeckungen von Robert Hooke und Antoni van Leeuwenhoek aus den gewohnten Fugen geraten [31, 40].

**Lemuel Gulliver** beginnt seine Laufbahn mit einer durchaus hohen Meinung von sich selbst. Er hält sich für äusserst talentiert und umfassend gebildet, dann bleibt aber der berufliche Erfolg aus, seine sozialen Bindungen sind unsicher und die Unruhe treibt ihn aufs Meer. Beschrieben wird also ein Fall der seit Odysseus bekannten *maritimen Poriomanie*, der Wandersucht zur See.

Zwischenlandungen in England sind von kurzer Dauer. Ankunft und Abfahrt erfolgt wiederholt aus den südenglischen *Downs* (Tab. 1). Psychosozial erfährt er auf seinen Reisen eine vierstufige Demütigung:

- er ist (relativ) gross, wird aber von Winzlingen überwältigt (Abb. 1);
- er ist tüchtig und wird dennoch von richtigen Riesen in die Tasche gesteckt;
- er ist gescheit, aber die Geistesmenschen nehmen ihn kaum wahr (bei ihnen könnte es sich allerdings auch um die Patienten einer Anstalt handeln; Abb. 2);
- er ist ein Homo sapiens, aber die edlen *Houynhnhmns* verweisen ihn als *Yahoo* des Landes.

Seine Identität wird erschüttert und schwindet, auch weil er sich zuletzt den Pferden angebiedert, dabei die Europäer und seinen Beruf verraten, sich selbst aufgegeben hat. Heimgekehrt verhält er sich wie ein Riese, oder er trabt oder spricht zuletzt wie ein Pferd. Tiefenpsychologische Deutungsversuche (*Master Bates* etc.) seit Ferenczi und Greenacre wurden im übrigen philologisch eingeordnet und abgewehrt [33, 73, 74].

Wäre es weniger wahrscheinlich, dass organische Faktoren bei entsprechender Symptomatik eine wesentliche Rolle spielten (Abb. 3), könnte man mutmassen, Swift/Gulliver habe neben einer Reihe von Positivsymptomen lange vor Eugen Bleuler [6] vier Kernsymptome der Schizophrenie dargestellt: die Ambivalenz der Gefühle bei den Lilliputanern; Affektstörung, Ängste bei den Riesen; Störungen der Assoziation auf Laputa; und den autistischen Rückzug nach dem Besuch bei den Yahoos und den edlen Pferden. Die seltsame Befremdung bei der Rückkehr empfanden jedoch auch Odysseus, Bligh, Robert Falcon Scott und andere [51].

### Jonathan Swift

Im Dunkeln bleibt die Position des Ich-Erzählers: beschreibt Swifts Gulliver, z. B. als auskunftswilliger Anstaltsinsasse, was er aus seiner Sicht und am eigenen Leib hilflos erlebt; der kunstfertige Autor Swift was er bei anderen auf der Straße und in Bedlam beobachtet? Zwischen den offensichtlichen Konfabulationen wirkt vieles zu treffend, zu authentisch um nur beliebig satirischen Zwecken zu dienen – wenngleich Swift sein Literatenleben lang skrupellos psychisch Kranke zum Zweck der Satire benutzt hat [90]. Manche behaupten, Swift sei selbst von Geburt bis zum Tod verrückt gewesen und zwar jenseits jeder medizinischen Diagnose [26]. Gulliver ist selbstverständlich eine überarbeitete Kunstfigur, dafür spricht schon sein Name [42], aber zugleich ist er eben auch Swifts Alter-Ego und dieser Swift hatte allen Grund für seine quecksilbrige Bitterkeit: der Vater vor seiner Geburt in Irland verstorben; die Mutter zurück in England; er vom Onkel in Dublin unter die Fittiche genommen; den *Magister Artium* nur per Gnadenerlass erhalten; politische Ambitionen in London frustriert; ins katholische Ulster strafversetzt zu einer anglikanischen Gemeinde mit ganzen 13 Seelen; wieder nach London und danach wieder nach Dublin, unglücklich verliebt usw. [14, 26, 27]. Ähnlich wird Gulliver ständig in fremde Welten verrückt und dies verarbeiten Gulliver und Swift auf ihre jeweils eigene Art [27, 82]. Das darf man dann in aller gallebitteren Rage ohne Rücksicht auf Kollateralschäden so schreiben. Von einer menschlichen Nähe oder Sympathie Swifts für psychisch Kranke ist nichts zu spüren; als Priester folgte er mit seinem Vermächtnis am ehesten einer moralischen Verpflichtung.

Aber Swift ging es auch um Krankheit und seine ganz eigenen Ängste. Er fürchtete schon lange vom Kopf her zu abzusterben und so kommt es schliesslich auch [27, 31, 57, 78]. Ab 1732 entwickelte er Schwierigkeiten mit dem Neugedächtnis; fühlte sich zwei Jahre später einsam, krank und deprimiert; zeigte ab 1735 ein vergröbertes Sozialverhalten und verlor ab 1736 an Gewicht. 1738 seit er nur mehr ein Schatten seiner selbst gewesen [5, 11, 92]. Er entwickelte was er als Autor vorweggenommen hatte [19, 56, 78]. Ab 1740 war er taub und aggressiv und 1742 erklärte man ihn für unmündig (*writ „de lunatico inquirendo“*). Nach seinem Tod im Jahr 1745 folgte die rückblickende diagnostische Einordnung zeittypischen Wandlungen [31, 57]. James Joyce meinte, Swift sei am Ende nur in den *Wald des Wahnsinns* entfleucht [43].
